# Real-time RT-PCR assay to detect Granada virus and the related Massilia and Arrabida phleboviruses

**DOI:** 10.1186/s13071-020-04110-5

**Published:** 2020-05-29

**Authors:** Laura Davó, Laura Herrero, Maria Paz Sánchez-Seco, Nuria Labiod, David Roiz, Elena Gómez-Díaz, Lourdes Hernandez, Jordi Figuerola, Ana Vázquez

**Affiliations:** 1 Centro Nacional de Microbiología, Instituto de Salud Carlos III, 28220 Madrid, Spain; 2 Departamento de Biología, Universidad Autónoma de Madrid, Madrid, Spain; 3 Estación Biológica de Doñana (EBD), Consejo Superior de Investigaciones Científicas, 41092 Seville, Spain; 4 Instituto de Parasitología y Biomedicina López-Neyra (IPBLN), Consejo Superior de Investigaciones Científicas, 18016 Armilla, Granada Spain; 5 Centro de Investigación Biomédica en Red de Epidemiología y Salud Pública (CIBERESP), Madrid, Spain; 6 MIVEGEC, Univ. Montpellier, IRD, CNRS, 34090 Montpellier, France

**Keywords:** Real-time RT-PCR, Granada virus, Massilia virus, Arrabida virus, Diagnosis, Surveillance

## Abstract

**Background:**

Granada virus belongs to the genus *Phlebovirus* within the Naples serocomplex and was detected for the first time in sand flies from Spain in 2003. Seroprevalence studies have revealed that Granada virus may infect humans with most cases being asymptomatic. Moreover, recent studies in vector samples revealed that the related Massilia and Arrabida phleboviruses could be also circulating in Spain. The objective of this study was to develop and assess a new sensitive real-time RT-PCR assay for Granada virus diagnosis able to detect the related phleboviruses Massilia and Arrabida.

**Methods:**

Two specific primers and one unique probe to detect Granada, Massilia and Arrabida viruses, without differentiating between them, were designed targeting the conserved L-segment of their genome. Sensitivity was assessed using 10-fold serial dilutions of quantified *in vitro* DNA samples. Specificity was evaluated by testing different genomic RNA extracted from other representative phleboviruses. The new assay was used for virus detection in sand flies collected in 2012 from the Balearic Archipelago, a touristic hotspot in the Mediterranean.

**Results:**

The real-time RT-PCR assay exhibited a sensitivity per reaction of 19 copies for Granada and Arrabida, and 16 copies for Massilia. No other related phleboviruses were detected. From the 37 pools of sand fly samples studied from four different Balearic Islands, we detected one positive in the island of Cabrera.

**Conclusions:**

To our knowledge, the method described here is the first real-time RT-PCR designed to detect Granada virus and the related Massilia and Arrabida phleboviruses. The study demonstrated that this is a rapid, robust and reliable assay for the accurate diagnosis of human infections as well as for virus surveillance in vectors.
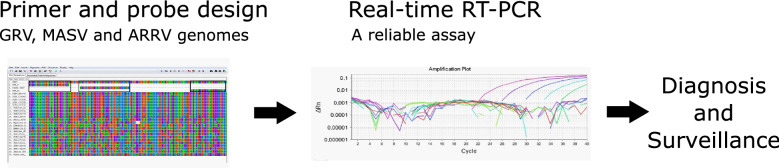

## Background

Sand fly-borne viruses of the genus *Phlebovirus* (family *Phenuiviridae)* are important emerging pathogens in Europe and are widely distributed [1]. All members of this genus are enveloped and the genome is composed of three negative RNA segments, S (small), M (medium) and L (large). The S segment encodes the nucleocapsid protein (N) and non-structural proteins (NSs); the M segment encodes two surface glycoproteins (G1 and G2) and non-structural proteins (NSm), and the L segment encodes the RNA-dependent RNA polymerase. The coding strategy seems to be common to all members of the family [2]. Until now, ten viral species defined by the sero-neutralization tests as well as several tentative species have been described [3], although their real medical importance has not been fully addressed yet. The sand fly fever Naples (SFNV) and Sicilian (SFSV) viruses, cause a “3-day fever”, characterized by flu-like symptoms, and Toscana virus (TOSV) may produce neuroinvasive infection (encephalitis and meningoencephalitis) [1]. TOSV is endemic in the Mediterranean countries, where the *Phlebotomus* vectors are present. Most TOSV infections have been reported in Italy, France and Spain and sporadically in Portugal, Greece, Cyprus, Turkey and Tunisia.

Recent reports suggest that other sand fly-transmitted phleboviruses may be involved in human disease [1, 4, 5]. This is the case for Granada virus (GRV), a phlebovirus detected for the first time in sand flies captured in 2003 and 2004 in the province of Granada (southeast Spain). Phylogenetic analysis of the complete genome revealed that GRV belongs to the *Sand fly fever Naples Complex* (SFNC) and it is probably a natural reassortant of the Massilia virus (MASV), donor of the L and S GRV genome segments, with a yet unidentified phlebovirus as donor of the M segment [6]. MASV was detected and isolated for the first time from *Phlebotomus pernicious* captured in 2005 in Marseille and Nice [7] and later from *P. perniciosus* captured in 2007–2008 in Portugal (Arrabida region) [8]. In the same study carried out in Portugal, a new phlebovirus named Arrabida (ARRV) was described [9]. ARRV was also phylogenetically classified as member of the SFNC showing a high nucleotide identity with MASV, GRV and Punique virus (PUNV). Whereas the sequences on the L and S segments of ARRV were closely related to MASV and GRV, the glycoproteins (M segment) were most closely related to PUNV, detected in sand flies from Tunisia in 2008, opening the question whether ARRV can be considered as a reassortant between MASV/GRV and PUNV [9]. Therefore, the closely related MASV, GRV and ARRV shared the L and S segments but differed for the M segment due to a reassortment process with different phleboviruses.

In Spain, a few studies conducted in sand flies have revealed the presence and co-circulation of several phleboviruses belonging to the SFNC (such as TOSV, GRV and ARRV) and *Salehabad virus species Complex* (such as Arbia virus). As mentioned above, GRV has been detected in Granada but sequences resembling the L-segment of GRV and MASV have been reported in other areas of Spain such as the Balearic Archipelago (Ibiza island) and Catalonia. However, without further sequence information for the M segment, they cannot be unequivocally assigned to GRV or MASV strains. Furthermore, similar sequences of viruses belonging to the *Salehabad virus species Complex* have been described in sand flies from Granada [6]. TOSV has also been detected in sand flies in Barcelona and Granada [6, 10]. In a recent study carried out in Madrid, six viral isolates were obtained from *P. perniciosus* captured in 2012 and 2013 [11]. The phylogenetic analysis and serological assays allowed the identification of two isolates of TOSV B genotype, three isolates strongly related to Italian Arbia virus (ARBV), and one isolate of a novel putative phlebovirus related to the recently characterized ARRV in South Portugal, tentatively named Arrabida-like virus.

Despite the increasing evidence that these viruses circulate in Spain, only human disease has been directly associated with TOSV in different regions (Murcia, Madrid, Catalonia, Andalusia and the Mediterranean coast) [12–14]. Two human serological studies carried out in Granada (Spain) have reported the GRV infective capacity. Collao et al. [6] revealed the presence of specific GRV-neutralizing antibodies in sera from healthy individuals collected in 2003 (10.8% of IFA positives, 1.8% of the total analyzed samples). In a different study, GRV seroprevalence was found to be 15.8% by IFA with 2.8% of neutralizing antibodies (18% of IFA positives). Moreover, anti-GRV IgM antibodies were detected (6.6%) in sera from patients with symptoms of unknown etiology [5]. Therefore, the authors concluded that GRV may infect humans but that most cases would probably be asymptomatic. Occasionally, mild GRV infections may occur, usually as self-limited febrile illnesses accompanied by other signs and symptoms (exanthema and/or acute respiratory infection) [5]. These infections most probably take place during the warm months of the year, which occurs with infections caused by other phleboviruses in Mediterranean countries. Whether GRV or the related MASV and ARRV could be circulating in other areas or countries and/or causing mild febrile diseases needs to be determined. Until now only a real-time RT-PCR designed in the nucleoprotein gene (S segment) has been published to detect MASV specifically [7].

All these data strongly support the need for new molecular assays that allow the specific detection of GRV, MASV and ARRV to improve current diagnostic methods and to be used in vector-borne diseases surveillance programmes for these phleboviruses. Therefore, the main objective of this study was to develop the first real-time RT-PCR assay able to detect GRV with high sensitivity, that at the same time might also detect the related phleboviruses MASV and ARRV. This assay will be very useful in studies about the possible implications of these viruses in human disease and in virus surveillance programmes.

## Methods

### Primer and probe design

Representative sequences from the three phleboviruses genomic segments (S, M and L) were obtained from the NCBI GenBank database and were aligned using the Clustal W program [15]. Primers and probes were designed using the Primer Express software v2.0 (Applied Biosystems, Foster City, CA, USA), to target a highly conserved region of the L segment of the GRV, MASV and ARRV genomes. We designed two different TaqMan probes with minor groove binder (MGB) and non-fluorescent quencher (NFQ) at the 3′-end, one for GRV-MASV-ARRV (Ph_Probe) and another for the internal control (IC_Probe), both of them were labeled with different fluorophores at the 5′-end (FAM and NED, respectively) (Table 1).

**Table 1:** Primers and probes used for Granada, Massilia and Arrabida detection

Name	Sequence (5′–3′)	Nucleotide positions
Primer_F	GAGCATGACATTAGCAGAGTTTCTGA	997–1022
Primer_Re	GTAGTCCCATTGCCAGCTTTCT	1102–1123
Ph_Probe	FAM-TGAGCTCTGATGAYATGTC-MGB-NFQ	1028–1046
IC_Probe	NED-CCAGCACACATGTGTCTACT-MGB-NFQ	

*Notes*: Positions are based on the complete sequence from GR25 virus (GenBank: GU135606)

*Abbreviations*: MGB, minor groove binder; NFQ, non-fluorescent quencher

### Construction of internal control plasmid and DNA standards

An internal control (IC) was used to exclude the presence of inhibition of the amplification step in the real-time RT-PCR (qRT-PCR). Overlapping primers IC_F and IC_Re were designed to construct the IC and a 68-bp DNA fragment was amplified by an end-point PCR. Primers designed in this technique were used to amplify part of the L gene of GRV (strain GR25; GenBank: GU135606). Due to the lack of reference standards for MAS and ARR viruses in our laboratory, DNA quantified standards were artificially constructed. For both viruses, 136-bp DNA fragments were obtained by an end-point PCR carried out with overlapping primers (Table 2). To obtain the IC and DNA standards, PCRs were performed using AMpliTaq DNA Polymerase with Buffer II (Applied Biosystems, Branchburg, NJ, USA) in a PCT-200, Peltier Thermal Cycler (MJ Research, Watertown, MA, USA), in a final volume of 50 μl, of a reaction mix containing: 5 μl 10× Buffer II, 2.5 mM MgCl_2_, 2.5 U of Taq enzyme, 0.5 μM of dNTPs (GE Health Care Life Science, Freiburg, Germany), 1 μM of each primer IC-F/IC-Re and RNAse-free H_2_O. PCR conditions were: 5 min 95 °C, 40 cycles of 30 s at 95 °C, 1 min at 72 °C, followed by a final extension step of 10 min at 72 °C. For the IC, the PCR product was purified and cloned using TOPO TA Cloning Kit (Invitrogen, MA, USA) according to manufacturer’s instructions and sequenced to confirm the absence of mutations. DNA concentration was determined spectrophotometrically measuring the optical density at 260 nm (OD_260_), using a NanoDrop instrument and 10-fold serial dilutions of the quantified DNAs were prepared in RNase-free H_2_O. Finally, for the IC, the plasmid was linearized using the *Not* I restriction enzyme. Known amounts of each DNA control were used for optimizing the conditions of the qRT-PCR.

**Table 2:** Primers used for internal control and Massilia and Arrabida positive controls construction

Name	Sequence (5′–3′)
IC_F	GAGCATGACATTAGCAGAGTTTCTGA**CCAGCACACATGTGTCTACT**
IC_Re	GTAGTCCCATTGCCAGCTTTCT**AGTAGACACATGTGTGCTGG**
MASV_F	TCAATAGAGCATGATATTAGCAGAGTTTCTGATTTCTTGAGCTCTGATGACATGTCTG**CTCTAAAGCCATCAGATGAATTGTAT**
MASV_Re	TGGTGTAGTCCCATTGCCAGCTTTCTCAGGTTGAAGTCTTTACTTAGTGGATT**ATACAATTCATCTGATGGCTTTAGAG**
ARRV_F	TCAATAGAGCATGACATTAGCAGAGTTTCTGACTTCTTGAGCTCTGATGATATGTCTA**CTTTGGTGCCATCAGATGGGTTATATA**
ARRV_Re	TGGTGTAGTCCCATTGCTAGCTTTCTCAGGTTGAAGTCTTTACTTAGTGGAT**TATATAACCCATCTGATGGCACCAAAG**

*Notes*: Complementary sequences are shown in bold for MASV and ARRV primers. For IC the sequences in bold correspond to the sequence of the IC probe

### Real-time RT-PCR assay

The real-time RT-PCR (qRT-PCR) was carried out using a commercial kit (Quantitec multiplex RT-PCR Kit; Qiagen, Hilden, Germany). For the assay, 5 μl of RNA sample was mixed with 20 μl of a reaction mix containing: 12.5 μl of 2× Buffer, 1.2 μM of each primer, 0.4 μM of virus probe, 0.2 μM of IC probe, 10 copies of IC plasmid, 0.3 µl of RT mix and nuclease-free water to reach the final volume of 25 µl per tube. Amplification conditions consisted of an initial retrotranscription step of 30 min at 50 °C, followed by an incubation step at 95 °C for 15 min, and 45 cycles of 45 s at 95 °C and 1 min at 60 °C. qRT-PCR was carried out in a 7500 Sequence Detection System from Applied Biosystems. The fluorescence emitted for FAM and NED was measured simultaneously at the end of each cycle. Quantification cycle (Cq) values were measured as the point at which the sample fluorescence signal crossed a predetermined threshold value.

### Sensitivity and specificity assessment

Sensitivity was determined according to the detection limit using the quantified DNA standards, which were diluted in the range of 10^7^–10^−1^ copies/µl and then tested by the qRT-PCR. Each concentration was assayed eight times in two consecutive days and the assay detection limit was calculated using the formula of Reed-Muench [16] for each virus. The analytical specificity is ensured by the thorough selection of the oligonucleotides (primers and probes), which were checked by sequence comparison analysis against publicly available sequences and evaluated by testing 20 different viral genomes from other phleboviruses. To determine the applicability of the assay for virus surveillance, insect samples collected in the field were tested.

### Study in field samples

Thirty-seven sand fly pools were collected in several Balearic islands in September 2012 using BG-Sentinel traps with BG-lure and carbon dioxide. The field work was focused on collecting hematophagous insects in Ibiza from 5th to 8th September (5 localities), in Formentera from 9th to 14th September (3 localities), in Cabrera from 15th to 20th September (4 localities) and in Dragonera from 22th to 24th September (4 localities) for a total of 269 trap/nights. Insects were identified and pooled according to species, sex, locality and date. Sand fly wings and body of individuals of the same locality and date were stored separately for molecular analysis. The head and genitalia were transferred individually into 1.5 ml Eppendorf tubes with 90% ethanol. For the identification, they were cleared in Marc-André solution (chloral hydrate/acetic acid) and mounted in chloral gum. Specimen identification was individually verified based on the morphology of the pharynges and/or the male genitalia or female spermathecae. In total 372 sand fly individuals were collected (6 in Ibiza, 105 in Formentera, 227 in Cabrera and 34 in Dragonera); these were divided into 33 pools of *Phlebotomus perniciosus* and 4 pools of *Sergentomyia minuta*. Pools were homogenized in 0.5 ml minimum essential medium supplemented with 10 % of fetal bovine serum and antibiotics (50 U/ml penicillin and 50 µg/ml streptomycin) before nucleic acid extraction. RNAs were extracted using QIAamp Viral RNA Mini Kit (Qiagen, Hilden, Germany) and analyzed for phleboviruses detection using a generic RT-nested-PCR [17] and specifically for GRV, MASV and ARRV using the new assay described in this paper.

## Results

### Design of primers and probes

Primers and probes were designed to target a highly conserved fragment of the L segment of GRV and the related MASV and ARRV amplifying a 126-bp sequence. An *in silico* test was performed using a partial nucleotide sequence alignment in the L-segment of representative phleboviruses to check the specificity and to identify possible cross-reactions with other different phleboviruses. After that, only one degenerate nucleotide was necessary to introduce in the phleboviruses probe sequence to detect the three viruses (Table 1). The amount of the IC included in the assay was fixed at 10 copies per reaction without affecting the qRT-PCR sensitivity.

### Viral RNA detection, qRT-PCR sensitivity and specificity

The detection limit was assessed using serial dilutions of the quantified GRV, MASV and ARRV DNA standards. Nineteen copies per reaction for GRV and ARRV and 16 copies for MASV were the detection limit estimated using eight replicates amplifying with a quantification cycle (Cq) value under 40 (Table 3). The standard curves and mean Cq values obtained in different assays indicated that the method is highly robust and reproducible.

**Table 3:** Limit of detection and data variation Cq of the qRT-PCR assay

Virus	copies/µl	*n*/*N* (%)	Cq-values	
			Mean ± SD	Range
GRV	0.1	0/8 (0)	ND	
	1	7/8 (86)	38 ± 0.4	38–39
	10	8/8 (100)	36 ± 0.5	35–36
ARRV	0.1	0/8 (0)	ND	
	1	7/8 (86)	37 ± 1.2	35–39
	10	8/8 (100)	34 ± 0.2	34–35
MASV	0.1	0/8 (0)	ND	
	1	8/8 (100)	37 ± 0.6	36–38
	10	8/8 (100)	34 ± 0.4	34–35

*Abbreviation*: n, positive results; N, replicates number; ND, not detected; SD, standard deviation

The qRT-PCR assay was found to be specific for the detection of GRV, MASV and ARRV without differentiating between them and no cross-reactivity was observed with any other sand fly borne phleboviruses (Table 4). The presence of phlebovirus genomes was confirmed using a generic RT-nested-PCR [17].

**Table 4:** Phleboviruses genome used in this study to test the specificity of the assay

Virus	Strain	GenBank ID	Origin	qRT-PCR result	Phlebo PCR result
Aguacate	VP 175 A	HM566138	Culture	–	+
Anhanga	Be An 46852	GU143711	Culture	–	+
Arbia	ISS PHL18	JX472400	Culture	–	+
Arumowot	AR 1284-64	GU143714	Culture	–	+
Bujaru	Be An 47693	GU143710	Culture	–	+
Candiru	Be H22511	GU143718	Culture	–	+
Chagres	JW10	GU143713	Culture	–	+
Icoaraci	BeAn24262	MK330768	Culture	–	+
Karimabad	I-58	GU143712	Culture	–	+
Pacui	Be An 27326	NC_043600	Culture	–	+
Punique			DNA standard	–	
Punta Toro	Balliet	KP272022	Culture	–	+
Rift Valley	Saudi 200-10911	DQ375401	QC OMS	–	+
Rift Valley	Kenya 83 (21445)	DQ375402	QC OMS	–	+
Rift Valley	200803167	JF311373	QC OMS	–	+
Sand fly fever Sicilian	Sabin	EF095551	Culture	–	+
Sand fly fever Naples	Sabin		Culture	–	+
Toscana	Issh Phl.3		Culture	–	+
Toscana	sch		Culture	–	+

*Notes*: Rift Valley genome was provided by an ENIVD quality control, quantified positive control of PUNV were specifically designed for this purpose, and the other phleboviruses belongs to the CNM virus collection

### Screening of phleboviruses in natural sand fly populations from Spain

**Fig. 1:**
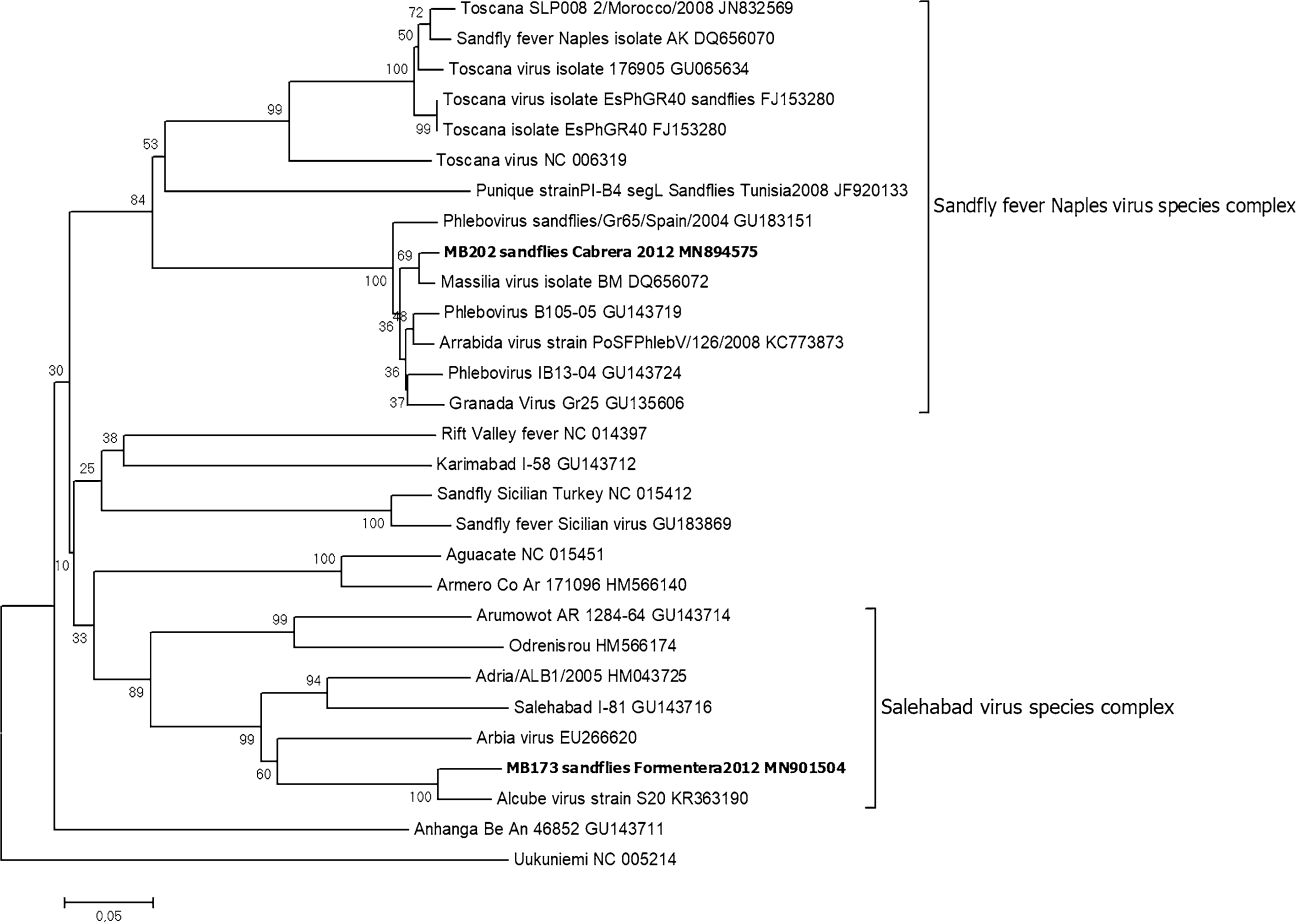
Phylogenetic tree based on 200 nucleotides of the partial L segment of the phleboviruses detected in the Balearic Islands (Spain). The phylogenetic tree was constructed using Neighbor-Joining method analysis with 1000 bootstrap replications. Sequence information correspond to virus, country detection, year, host and GenBank accession number. The sequences of viruses detected in this study are indicated in bold. The GenBank accession number are MN894575 (MB202) and MN901504 (MB173)

Thirty-three *P*. *perniciousus* pools and four *Sergentomyia minuta* pools were analyzed. These samples were previously analyzed using a generic RT-nested-PCR for phleboviruses developed by our laboratory that targets a 200-bp fragment of the L-segment. Two positive pools from *P*. *perniciousus* were detected, one in Cabrera (MB202) and the other one (MB173) in Formentera. The phylogenetic analysis of the sequences revealed that one sequence (MB202) is related to MASV, GRV and ARRV and the other one (MB173) is related to Alcube virus (Fig. 1). Using the novel qRT-PCR assay developed in this study, only one pool (MB202) was positive corresponding to the MASV-like pool detected with the generic RT-nested-PCR. An additional fragment of 903 bp of this sample was amplified in the M segment with an unpublished in-house RT-PCR, and the sequence obtained showed 83% of homology with MASV strain W (GenBank: EU725772) detected in sand flies from France. Based on these results, the qRT-PCR was shown to be specific and no false-positives were detected.

## Discussion

Phleboviruses are distributed across much of the Old World, including the Mediterranean basin. Recent investigations suggest that the diversity of this group of viruses is higher than initially suspected, but data on prevalence, geographical distribution and pathogenesis remain unknown [1, 8, 11, 18]. This could be due to a lack of sensitive and specific methods for virus detection and quantification, but also to the lack of studies in natural populations. This is the case of the related phleboviruses GRV, MASV and ARRV that, until now, have been detected in sand flies from Spain, France and Portugal.

In Spain, several phlebovirus species belonging to two antigenic complexes have been described in several geographical areas from the northwest, center and south of the country. Viruses belonging to the *Sand fly fever Naples virus species complex*, Toscana, Granada, Massilia-like and Arrabida-like have been detected, and Arbia-like virus belonging to the *Salehabad virus species complex* has been detected [5, 6, 11]. Amongst them, TOSV is the only member recognized to cause human disease. Whereas there is no clear evidence that MASV, GRV, ARRV or ARBV are pathogenic to humans, the last reports of seroprevalence studies carried out in humans in Spain, showed that GRV may infect humans with low prevalence, leading to asymptomatic or self-limited febrile illnesses cases accompanied by other signs and symptoms like exanthema and/or acute respiratory infection [5]. Therefore, despite there being very limited data about the capacity of the latter viruses to cause disease, this possibility cannot be neglected. Given the lack of data about these novel viruses and the possibility that they can infect and cause disease to humans, additional epidemiological and clinical studies on natural populations are strongly needed. Moreover, the viral loads reached in human infections by these viruses are unknown, and for this reason a highly sensitive PCR available for diagnosis is required. Until now, only one real-time RT-PCR designed in the S segment has been published to specifically detect MASV [7], but it has not been validated with the related GRV and ARRV phleboviruses which were described later. Furthermore, new molecular assays to detect these viruses are required that enhance sensitivity and specificity at the same time that are applicable to a broad range of samples.

In this study, we developed a rapid, sensitive and specific qRT-PCR for clinical investigation into GRV, MASV and ARRV infection. Here we show the applicability of a new qRT-PCR assay with enhanced sensitivity and broad range spectrum able to detect the three phleboviruses simultaneously and which we used to detect these pathogens in sand flies collected in the Balearic Archipelago, a touristic hotspot in the Mediterranean. Our findings demonstrate for the first time the detection of MASV-like in *P. perniciousus* from Cabrera island (Balearic Archipelago), which would support the spread and circulation of this virus in Spain and in neighbouring Mediterranean countries.

## Conclusions

The new method developed here is a rapid, robust and reliable assay for the accurate diagnosis of human infection as well as for surveillance in vector populations, providing information about the disease burden of these infections.

## Data Availability

All data involved and arising from the study are included in this published article.

## Abbreviations

SFNV: Sand fly fever Naples virus
SFSV: Sicilian fever Naples virus
TOSV: Toscana virus
GRV: Granada virus
SFNC: Sand fly fever Naples complex
MASV: Massilia virus
ARRV: Arrabida virus
PUNV: Punique virus
ARBV: Arbia virus
MGB: minor groove binder
NFQ: non-fluorescent quencher
IC: internal control
qRT-PCR: real-time RT-PCR
